# Anthropogenic Volatile Organic Compound (AVOC) Autoxidation as a Source of Highly Oxygenated Organic Molecules (HOM)

**DOI:** 10.1021/acs.jpca.1c06465

**Published:** 2021-10-07

**Authors:** Matti Rissanen

**Affiliations:** Aerosol Physics Laboratory, Physics Unit, Faculty of Engineering and Natural Sciences, Tampere University, 33720 Tampere, Finland

## Abstract

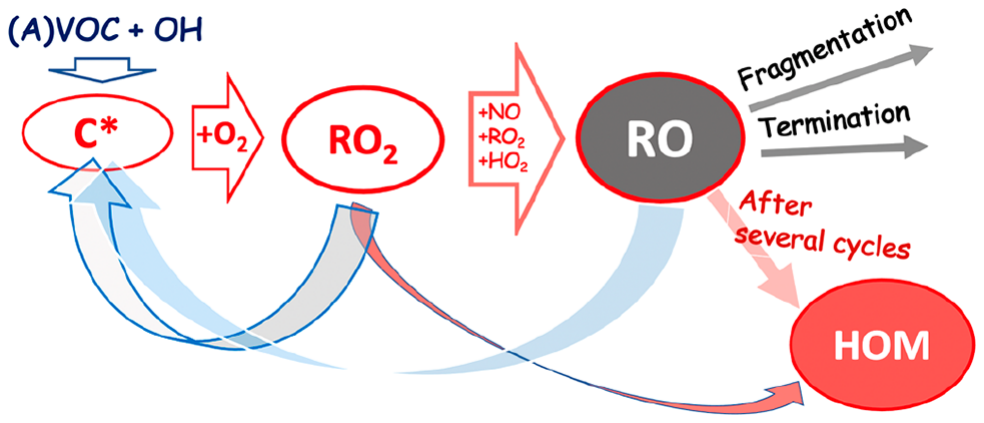

Gas-phase hydrocarbon autoxidation is a rapid pathway for the production of in situ aerosol precursor compounds. It is a highway to molecular growth and lowering of vapor pressure, and it produces hydrogen-bonding functional groups that allow a molecule to bind into a substrate. It is the crucial process in the formation and growth of atmospheric secondary organic aerosol (SOA). Recently, the rapid gas-phase autoxidation of several volatile organic compounds (VOC) has been shown to yield highly oxygenated organic molecules (HOM). Most of the details on HOM formation have been obtained from biogenic monoterpenes and their surrogates, with cyclic structures and double bonds both found to strongly facilitate HOM formation, especially in ozonolysis reactions. Similar structural features in common aromatic compounds have been observed to facilitate high HOM formation yields, despite the lack of appreciable O_3_ reaction rates. Similarly, the recently observed autoxidation and subsequent HOM formation in the oxidation of saturated hydrocarbons cannot be initiated by O_3_ and require different mechanistic steps for initiating and propagating the autoxidation sequence. This Perspective reflects on these recent findings in the context of the direct aerosol precursor formation in urban atmospheres.

## Introduction

Air pollution shortens the lifespan and decreases the well-being of an increasing number of lives.^1−5^ Especially airborne particulate matter is of great concern.^6,7^ The situation is worse in the megacities across the globe, where more people are living than ever before.^8,9^ While the limiting of emission sources is the most efficient way to mitigate air pollution, a large fraction of the emissions will remain unavoidable. Part of the inability to hinder the problem raises from our incomplete understanding of volatile organic compound (VOC) oxidation in the gas phase. Perhaps most alarmingly, the formation of particulate air pollution from gaseous sources is particularly poorly understood.

Recently the rapid gas-phase autoxidation of endocyclic alkenes initiated by a reaction with ozone (i.e., ozonolysis) was shown to yield highly oxygenated organic molecules (HOM).^10−12^ HOM were originally found in the Finnish Boreal forest^10,13,14^ and were promptly connected to the “missing organic compounds” expected to explain organic aerosol measurements.^15−17^ Consequently, they were shown to be the key compound class involved in the formation and growth of atmospheric secondary organic aerosol (SOA) mass.^10,18^ The current understanding of HOM was recently reviewed by Bianchi and co-workers^19^ in order to synthesize the decade of observations into a more meaningful format. They defined HOM as species that are (i) formed via a sequential autoxidation of peroxy radicals (RO_2_) (ii) under atmospherically relevant conditions and (iii) contain six or more oxygen atoms. This definition contains the gas-phase aerosol precursors formed by a rapid sequential oxidation through consecutive H-shift reactions, while also excluding common plant-synthesized, highly oxygenated carbohydrates (e.g., sugars). The HOM contain several hydrogen-bond-donating functional groups such as alcohols and hydroperoxides and aggregate by a peroxide “dimer” formation, and their general stability on surfaces and in condensed phase media is still largely in doubt. This strict definition currently leaves out certain important aerosol forming molecules with similar properties in clustering and condensing reactions, such as multicarboxylic acids (e.g., citric acid and 3-methyl-1,2,3-butanetricarboxylic acid [MBTCA]), which do not have known fast gas-phase formation routes. Also, the oxygenated compounds left out from the HOM definition (e.g., multicarboxylic acids and polyalcohols) are considerably more stable in condensed phase, and while they have a similar propensity for hydrogen bonding and clustering reactions, it is possible that they do not react significantly in the condensed phase.

**Figure 1:**
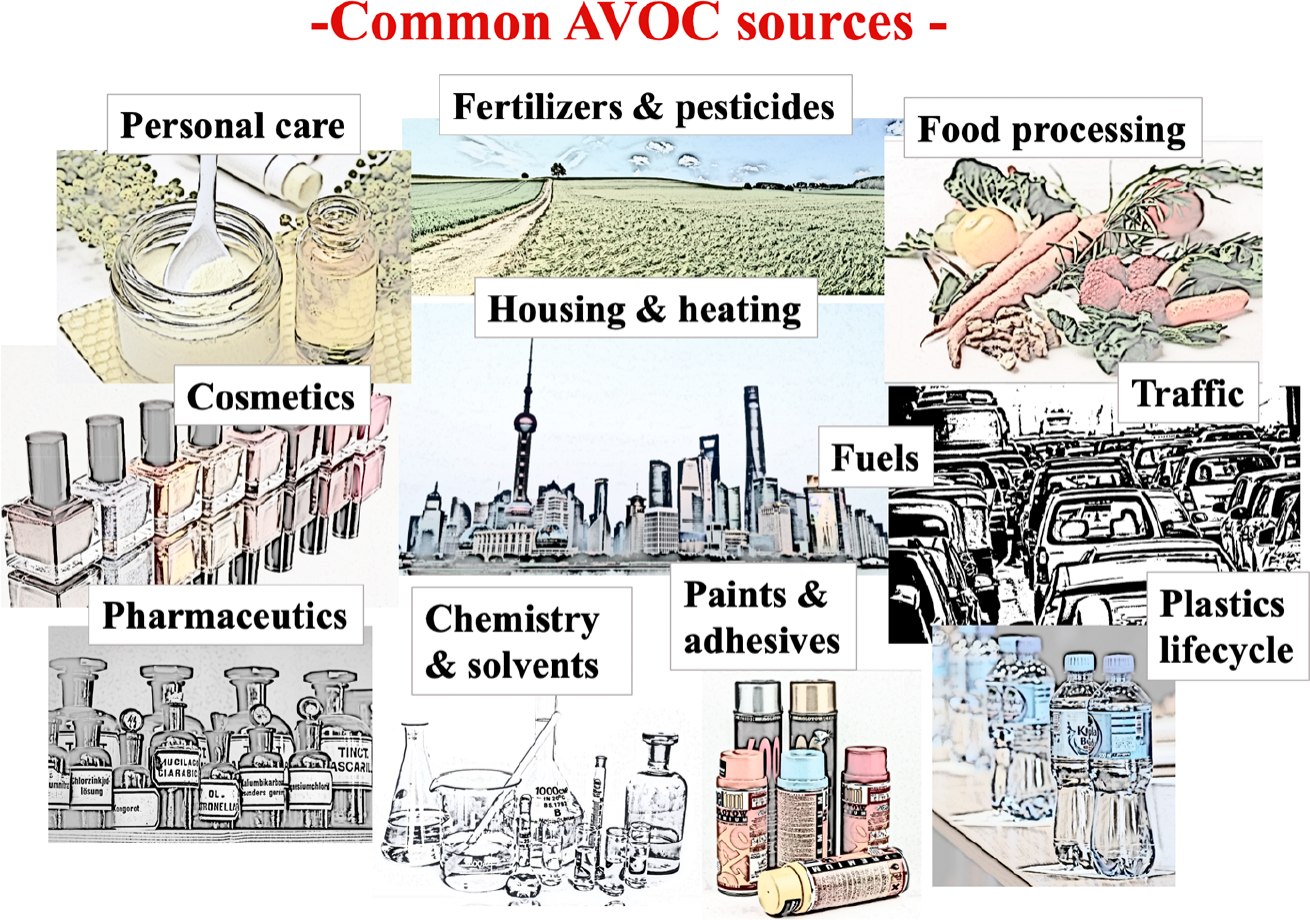
Examples of common AVOC sources in our living environments.

Anthropogenic volatile organic compounds (AVOC) are hydrocarbons released by human activities. They are emitted from countless activities of the society, such as traffic, energy production, industry, from solvents, adhesives, paints, lubricants, and wear-reducing products, from cosmetics and personal care-products, and so on, and so on (Figure 1).^20−22^ In practice, AVOCs cover all hydrocarbon subclasses containing all functional groups from aromatic to aliphatic compounds. In fact, the division to AVOC and BVOC (i.e., biogenic VOC) is chemically completely arbitrary; often, the same compounds are emitted by both anthropogenic and biogenic processes (e.g., isoprene [CH_2_=CHC(CH_3_)=CH_2_] and ethylene CH_2_=CH_2_^21,23−25^). Generally, AVOCs are thought to consist mainly of aromatic compounds and alkanes, and common for these species is that they do not react appreciably with O_3_. This more chemically rigorous classification of AVOC is adopted here too to allow for an easier discussion, notwithstanding the shortcoming that several common anthropogenic compounds do not fit in (e.g., ethene, propene, and acetylene^21^). The ozonolysis of double bonds is an important initiation step in the formation of atmospheric particulate matter, and thus a division of compounds that either react or do not react with O_3_ is useful. Moreover, most of the information we currently have on HOM formation has been gained through ozonolysis oxidation experiments.

The structural analogies of aromatic AVOCs to several endocyclic BVOCs together with their known high SOA yields motivated early experiments on AVOC HOM formation.^26,27^ Aromatic systems have double bonds coupled to cyclic structures (although still no appreciable reaction with O_3_), and the pathways up to five oxygen-containing bicyclic peroxy radicals (BPR) are well-established (see, e.g., refs (28−30)). Subsequently, several aromatic systems were shown to generate HOM with a high yield, in line with their reported high SOA yields. In stark contrast, saturated acyclic alkanes are completely devoid of these sites known to enable a gas-phase autoxidation propagation by sequential H-shift reactions under atmospheric conditions but still are reported to generate a significant SOA mass in chamber oxidation experiments.^26,31,32^ Thus, the recent finding of alkane HOM formation by Wang et al.^33^ seemingly contradicts our basic understanding of gas-phase organic oxidation chemistry,^34,35^ yet is fully in line with the view of HOM being the pathway to atmospheric SOA. This is understandable, as the same principles govern the atmospheric autoxidation sequence in any VOC system, the loosening of C–H bonds due to adjacent and nearby functional groups,^11^ or by heat.^33^ In pure, naked hydrocarbons the initiation of RO_2_ H-shift reactions is very slow,^36^ but with a functionalization of the molecule, or at elevated temperature, the initiation considerably speeds up.^33^

The detailed pathways to HOM in aromatic compounds remain far from understood, notwithstanding the relatively many publications already devoted to the topic. Currently only Wang et al.^37^ have inspected the first steps of alkylbenzene-derived RO_2_ H-shifts by detailed quantum chemical computations, whereas others have mainly reported product compositions and overall HOM yields.^38−41^ It seems that AVOC HOM from both aromatic and alkane sources have escaped observations due to the same reasons HOM were not found in the first place: (i) The yields of HOM generally fit into common measurement uncertainties (i.e., HOM yield ∼0.1–10%^19^), (ii) the sampling from experimental setups has not been optimized for free radicals and sticky aerosol precursors (i.e., HOM were lost to the walls), and (iii) oxidation studies and mass spectrometric detection are performed at reduced pressure and low O_2_, decreasing, or even preventing, the formation of HOM.

This perspective concerns AVOC HOM formation by autoxidation with a focus on the recent advances in our understanding and on the remaining challenges in uncovering the underlying mechanistic steps leading to these in situ aerosol precursors. The text is structured as follows. First the AVOC HOM reported in recent literature is presented, followed by the related challenges in instrumental detection methodologies, field observations, and theoretical computations. Then the mere lack of chemical intuition in finding the correct reaction sequences is detailed and discussed, with the last chapters dedicated to an examination of the burning questions that are difficult, or even impossible, to answer with the current research methodologies.

## AVOC HOM in the Literature

The current literature of AVOC HOM formation is presented in Table 1. Aromatic systems have received the most attention with benzene and several alkylbenzenes being studied experimentally and theoretically by Wang et al.,^37^ a collection of compounds including polyaromatics by Molteni et al.,^38^ the sequential and auto-oxidation of benzene and HOM formation in several other systems by Garmash et al.,^39^ toluene and 1,2,4-methylbenzene by Zaytsev et al.,^40^ and the photo-oxidation of toluene and naphthalene by Wang et al.^41^ Numerous earlier works have investigated the formation of bicyclic peroxy radicals that are the starting material for HOM formation from aromatics (e.g., refs (28−30)) yet have not reported any HOM products. Currently, only Wang et al.^33^ have reported alkane-derived HOM, though several papers have described important steps on the way to HOM formation.^36,42−45^

**Table 1:**
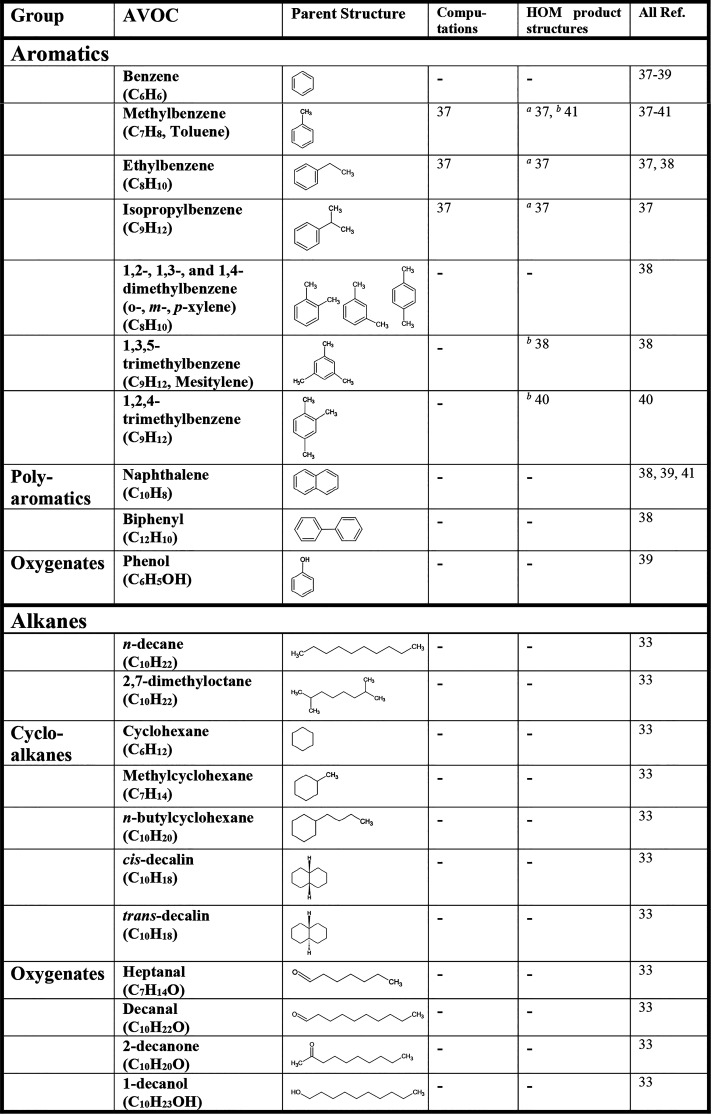
AVOC HOM in the Literature

a Computed product pathways.

b Proposed pathways based on previous literature.

**Figure 2:**
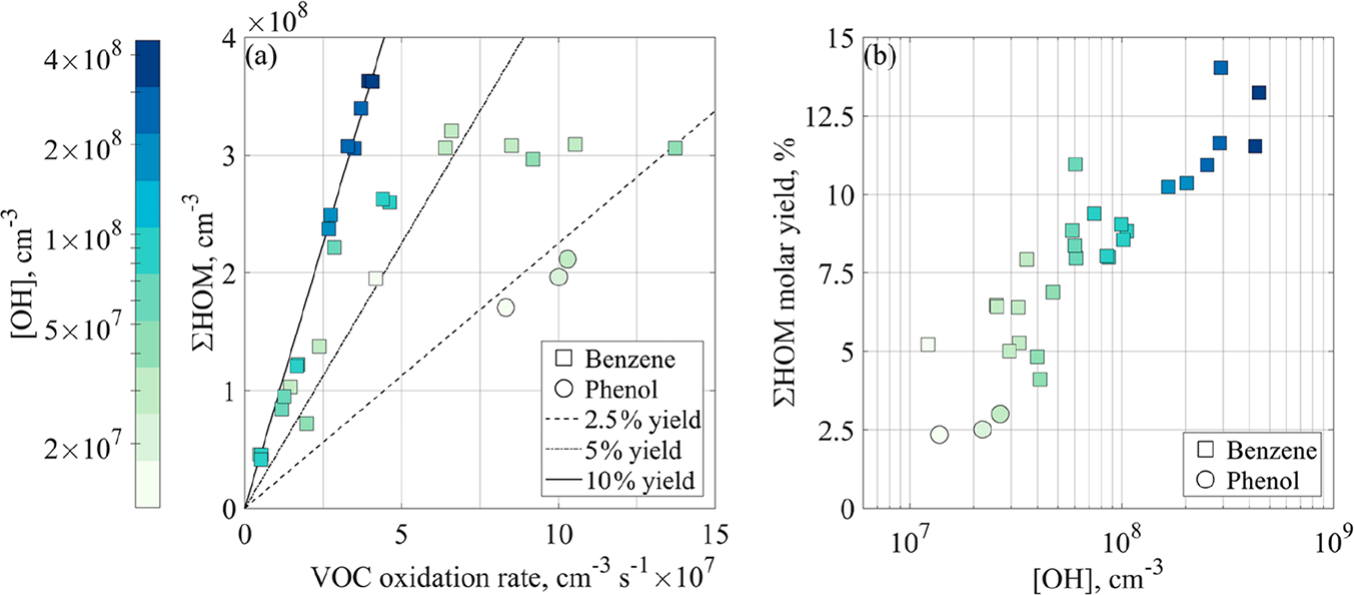
HOM concentrations (a) and yields (b) observed in benzene (squares) and phenol (circles) OH oxidation experiments. The dependence of benzene HOM on OH and VOC oxidation rate uncovers the multigeneration oxidation in competition with autoxidation through BPR. The color represents the concentration of hydroxyl radicals. Reprinted with permission from ref (39). Copyright 2020 Copernicus Publications.

While the amount of different AVOC systems investigated to date is already considerable, the detailed mechanisms for HOM formation has not been obtained for any of them. In the more studied aromatics, steps beyond BPR remain highly uncertain. An important complexity arises from a similar formation of gradually oxidized species by accelerating multigeneration oxidation (i.e., oxidation initiation, termination, and again initiation) and was very recently inspected by Garmash et al.^39^ (Figure 2; also noted in previous works, e.g., refs (27) and (46)). The OH reaction rate coefficients of the primary oxidation products are considerably faster than the parent rates, with a concomitant higher probability in initiating autoxidation, as the oxidized functional groups ease the subsequent H-shift reactions. Moreover, the isomeric products are not separable with the current mass spectrometric techniques applied for HOM detection and, thus, pose severe challenges for experimental product determinations, which are discussed next.

## Challenges in Detection

All direct observations of gas-phase HOM have been obtained with atmospheric pressure controlled chemical ionization mass spectrometry (APcCI-MS; also referred to with other names in the literature such as selected ion CI-MS^47^). Most of the studies have applied selective adduct-forming negative polarity reagent ions for a sensitive detection of a wide variety of HOM. Especially the utilization of nitrate ion (NO_3_^–^)-based charging has been instrumental for the original discovery and subsequent insights (e.g., refs (10, 11), and (48)). Recently, also the usefulness of positive polarity adduct formation has been demonstrated by Berndt et al.^33,49^ This very sensitive and selective, controlled chemical analysis technique should not be confused with the rough APCI-MS methods commonly used for detection in conjunction with a chromatographic separation. The extreme selectivity of cluster formation in the ion–molecule reactions of APcCI charging means that the mass spectrum can be obtained almost devoid of chemical noise, resulting in an extremely sensitive detection of the HOM.

In APcCI methods either a single reagent ion (e.g., iodide and bromide^50−52^) or a collection of reagent ions (e.g., nitrate and acetate^51,53^) are produced in a controlled manner at atmospheric pressure in a separate ion production unit, after which the reagent ions and the targeted sample are mixed with the help of electric fields in an ion–molecule reaction region. Subsequently the charged sample is drawn into the mass spectrometer through an orifice or a capillary, with practically no dilution, as the charging happens before the entrance of the vacuum chambers. The careful control over the ion pool that meets the sample improves the sensitivity and reduces the secondary chemistry tremendously. Also other reagent ion introduction designs have been implemented; for example, Sipilä et al.^54^ used sulfuric acid injection directly into the sample flow, to induce HSO_4_^–^ formation in reactions with NO_3_^–^, followed by its clustering with the target molecules. The controlled manner by which well-chosen ions can be produced together with the extreme selectivity of the clustering reaction make the APcCI currently an unrivaled method for gas-phase HOM detection.

Novel proton-transfer-reaction mass spectrometers (PTRMS) work with largely the same principles as the APcCI-MS, and certain instrumental configurations have shown promising developments in the detection of HOM.^55^ However, PTRMS reaches inherently lower detection sensitivities (i.e., higher detection limits) due to low-pressure ionization sources (i.e., most of the sample is lost before the sample is ionized) and due to nonselective ionization schemes, making charge availability problematic. Nevertheless, with the novel type of ion transfer optics, they have been shown useful in recent HOM research and, with further optimization, could become of great importance. Especially with several carefully chosen proton-transfer schemes and the proton transfer being performed at atmospheric pressure could allow for a selective detection of both the reagents and a wide array of products with a single instrument. Yet, this type of an approach remains to be demonstrated in atmospheric research.

Without a doubt, the community would benefit from more instrumentation capable of detecting these crucial, low-volatile, and reactive aerosol precursors in situ. Arguably, the HOM research community suffers from this scarcity of detection methods, and some have even criticized HOM to be merely an artifact of the used instrumentation.^56^ Yet, the growing number of studies probing HOM and subsequent new-particle and SOA formation in a laboratory and in the field, and the increasing variety of reagent ion chemistries used in HOM detection, are slowly unifying the perception of HOM importance in gas-phase particulate matter formation.

In contrast to the applied mass spectrometric detection methods, spectroscopic and chromatographic techniques could supply direct information on the product structure and would be highly beneficial for inferring the presently uncertain HOM structures. However, their inherent experimental limitations prevent a utilization of their benefits. In spectroscopic techniques, the spectral overlaps of similar functional groups within similar chemical neighborhoods constitute a tremendous problem for speciation. On the one hand, the less-oxidized products and intermediates present at higher concentrations than HOM will contain the exact same functional groups in the exact same molecular positions (i.e., they are gradually oxidized products from the same parent compound oxidation), maximizing the spectral overlaps. In chromatographic techniques, on the other hand, the transfer limitations of the elution columns generally prevent the highly functionalized and polar HOM to pass through. Additionally, the high surface activities and reactivity of polyperoxide HOM mean that, if they can travel through the chromatographic column, they are unlikely to do it without a change in their molecular structure. Moreover, HOM completely lack authentic standards, and, as a currently fully gas-phase species, their stability in condensed phases is a mystery. The HOM standards would have to be formed in situ with a controlled gas synthesis, a chore that is in practice close to impossible to accomplish without a multitude of secondary unwanted reaction products.

While the chromatographic and spectroscopic techniques would be ideal in solving detailed HOM structures, their successful application requires a surmounting of these technical challenges first. Recently, gas chromatography has been used for the quantification of several multifunctional aromatic oxidation products, which are the likely prestages of HOM formation through a sequential oxidation.^40,46,57^ If the technical limitations can be overcome, then a combination of chromatographic separation and spectroscopic detection would provide a currently unrivaled structural characterization for the HOM, yet nothing like this seems available in the foreseen future. Another fundamental technical obstacle comes from the detection limit. Individual HOM compounds are present from below 10^5^ cm^–3^ to up to ∼10^8^ cm^–3^ gas-phase concentrations even in optimal laboratory settings. Such minute quantities present challenges for any detection methods notwithstanding the nature of the target material. Thus perhaps a more fruitful approach is to develop chemical ionization mass spectrometry toward a structural analysis methodology.

While mass spectrometers are generally far from ideal at extracting three-dimensional molecular information, they are able to resolve features of the molecular structure. Several methods can be used to infer a deeper insight into the HOM construction. D_2_O-mediated H to D exchange,^11^ ion-mobility spectrometry (IMS),^58^ and MS-MS fragmentation experiments^59^ have all been used to verify the assumption that several HOM isomers are formed from the oxidation sequences. If single-oxidation pathways and their subsequent products could be isolated, then a threshold photoionization (e.g., with vacuum ultraviolet^60^ or multiphoton infrared radiation^61^) and potentially other compound-specific characteristics could be used for a molecular identification. However, currently it seems that only in very specific cases a single oxidation pathway could be responsible for the HOM formation, and generally isomers are formed. For an understanding of the chemistry the most important isomers to distinguish would be those that are susceptible to a very different subsequent chemistry, for example, double bonds versus cyclic structures and bridged oxygens (e.g., epoxide and endoperoxide) versus carbonyls, which hinder and enhance the oxidation propagation in very specific ways.

As yet unexplored, structural information on oxidation products could also be inferred indirectly by utilizing several well-chosen reagent ion schemes. Only a certain type of reagent ions bind to certain functional groups, and thus, a chemical filtering of the product distribution becomes possible.^50,51,53,62−64^ For example, distinguishing between carbonyl and alcohol groups with a reference to molecular composition from the MS measurement. In such a technology, a rapid application of several ionization schemes would be highly beneficial. The recently developed multischeme chemical ionization inlet (MION^50^) type of approach would allow this, enabling the rapid switching between multiple reagent ion schemes in both ion polarities. Even the utilization of several concomitant proton-transfer schemes could be envisioned, enabling an overcoming of the charge balance problem mentioned above.

**Figure 3:**
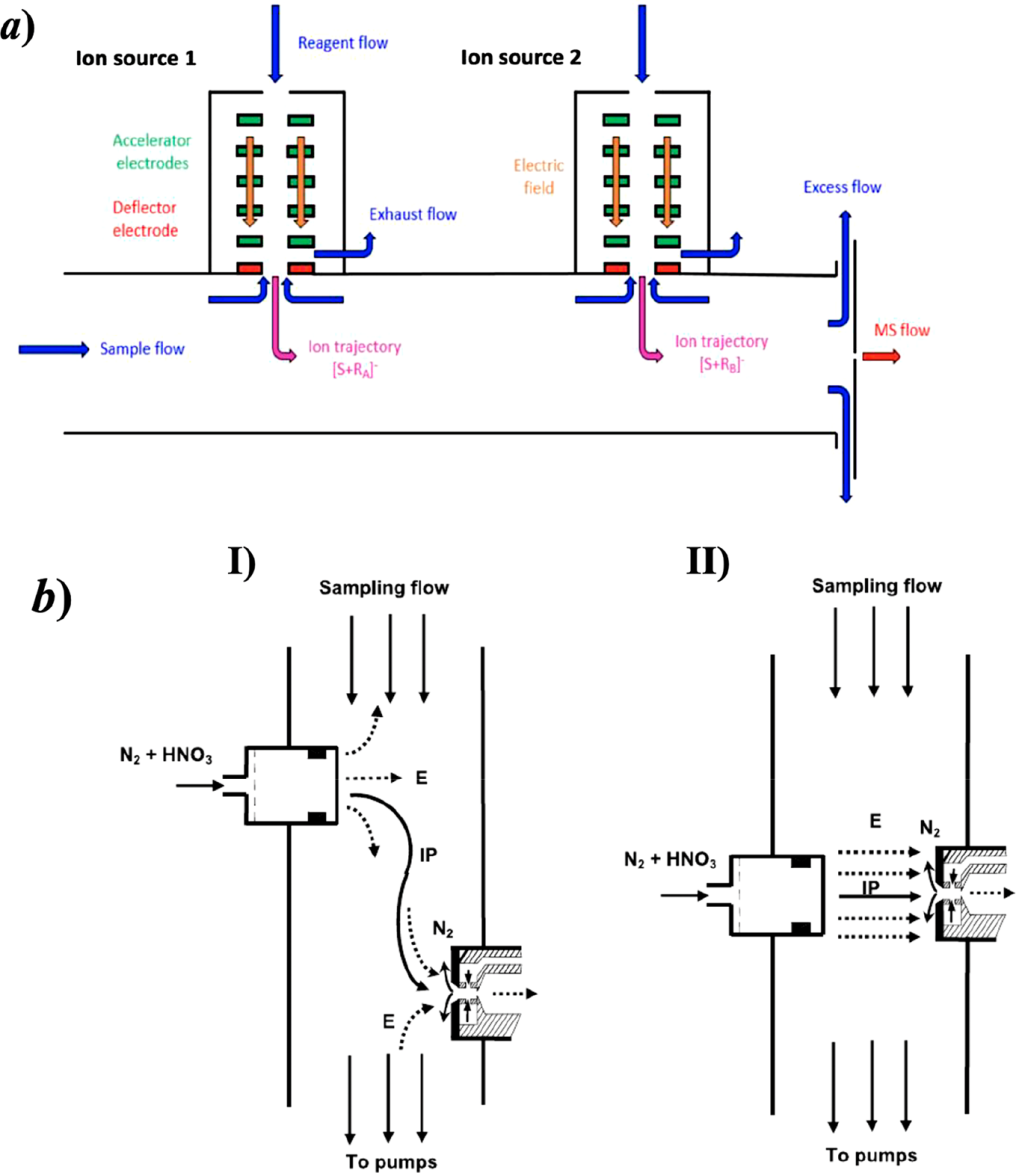
Two examples of novel chemical ionization inlet designs that allow an investigation of the rapid HOM formation processes in an AVOC oxidation. (a) MION with two ion injectors, the source 2 providing the short ionization time. (b) The “cluster-CIMS” in (i) flow tube mode and in (ii) transverse ion mode, the latter geometry being suitable for the short reaction time experiments. (a) Reprinted in part with permission from ref (50) (copyright 2019 Copernicus Publications). (b) Reprinted in part with permission from ref (67) (copyright 2010 American Geophysical Union).

Currently the HOM structures are only known by inference to computed HOM formation mechanisms. In experimentally detailing these mechanisms, time scales are crucial yet problematic. The isomeric complexity likely rapidly increases with reaction time, and at short times only the fastest pathways have reached to highly oxidized products. This is amply exemplified in recent research on a-pinene HOM formation.^59,65^ The a-pinene product distribution considerably changes from subsecond to tens of seconds reaction time. The endoperoxide bridge reportedly important for the rapid early oxidation propagation seems to be absent already at 30 s reaction time according to the MS-MS analysis. Similar changes in peroxy radical distributions as a function of reaction time in isoprene oxidation have been discussed by Wennberg et al.^66^ While the long reaction times are rather trivial to obtain when larger reactor setups are utilized, the short reaction times pose considerable challenges for the CIMS research methodology. CIMS is poorly suited for the study of rapid chemical changes due to the finite time needed to charge the sample by ion–molecule reactions. Especially the inlets optimized for a sticky and reactive reaction product detection (e.g., several inlets that mimic the pioneering work by Eisele and Tanner^47^) have sheath flows and characteristic long charging times. The faster CI-inlets, the MION working by ion injection,^50^ and the “cluster CIMS” with a transverse ionization geometry by Zhao et al.^67^ are more suited for this type of research (Figure 3). Yet even more direct, and faster, detection would be preferable and potentially available by a direct photoionization, but this has not been demonstrated yet.

Thus, it is the all-important characteristics of the APcCI-MS methodologies that have offered the recent leaps in HOM detection. The selectivity, accompanied by the inherent sensitivity, combined with the practically wall-less sampling under normal atmospheric conditions. Most of the classical information on oxidation pathways comes from studies performed at low pressures, especially the related MS work, which partly explains why the discovery of atmospheric autoxidation and the subsequent HOM formation took us so long. Considering all the experimental challenges in resolving the HOM chemistry, it seems clear that the APcCI techniques will remain the sole detection methodology for HOM still for some time.

## Challenges in Field Observations

The challenges in lab observations become only worse in the field. Therefore, it is needless to say that a quantification of individual AVOC-derived HOM from the ambient gas-phase has not been demonstrated. Their largely unknown molecular structures and, thus, unknown detection sensitivities, combined with a diversity of chemicals leading to HOM with similar compositions, will continue to make this task extremely challenging. Fortunately, HOM formed by similar autoxidation sequences are likely to share similar enough molecular properties that their quantification as a group of compounds is still worthwhile for an understanding of SOA budgets (i.e., HOM have low to extremely low volatility^18^ and contain hydrogen-bonding −OH and −OOH groups that enable clustering and tangling to a substrate^51,68^). Thus, often it is likely enough to merely estimate the sum of HOM from the mass spectrometer signal from products with HOM compositions. This carries some extra uncertainty due to the varying detection sensitivities for molecules with different functional groups, yet the difference in charging efficiency is likely relatively small for such a highly oxygenated reaction products.^51^ Furthermore, it is rather easy to propose HOM compositions based on simple mechanistic rules concerning their formation pathways. For example, if an OH H-abstraction reaction started the oxidation sequence and the only bimolecular steps were O_2_ additions, then likely a succession of products with varying, high oxygen content, the same amount of C atoms, and two fewer H atoms are seen, the second H being lost during an −OH ejection after an oxidation-terminating H-shift from a hydroperoxide C. However, in reality every branching point, especially C–C bond scissions and H-atom scrambling, complicate this task, leading to a wider array of possible product molecular structures. However, currently most of the observed HOM products pertain to certain oxidation propagation rules (e.g., refs (19) and (69)), with few C atoms lost on the way.

Most likely places for AVOC HOM observations are polluted urban atmospheres and places in close proximity to industrial activities and combustion sources. These environments are characterized by a vast number of different emitted chemicals and airborne particulate surfaces where HOM can deposit on. Generally urban atmospheres can have high AVOC HOM concentrations as exemplified recently by McDonald et al.^20^ in considering the vast amount of different petrochemically derived volatile chemical products (VCP) emitted from the daily routines of the society.

## Challenges in Computation

Most of the information on HOM formation mechanisms and molecular structures have been obtained by quantum-chemical computations.^19^ The inherent complication in this research is a result of the rapidly growing number of potentially relevant unique molecular structures that contribute to the important reaction steps. Often it is not a simple task to decide which pathway to follow computationally, and the previously generated rules of thumb slowly lose their meaning as a function of molecular complexity. The substituent effects of functional groups that affect the H-transfer rates are also not necessarily additive. This rapidly increases the number of species to consider, and an even larger number results from their mutual reactions—often being referred to as the combinatorial problem of organic chemistry. For example, isomers (i.e., molecules with the same functional groups but distributed differently) are almost certainly also reacting differently, yet even the conformation of the molecule (i.e., the same molecular structure but different spatial structure) can have a strong influence on its subsequent chemistry.^42^

A coupled technical hurdle comes from the fact that several of the key reactions require state-of-the-art computational tools, which consume a vast amount of computational resources. For example, recently the decades old mystery of gas-phase “dimerization” of peroxy radicals into oxygen-bridged peroxides,^70−72^ which have special importance in growing atmospheric SOA,^48,73,74^ was resolved only by an application of far more sophisticated computational methodology (i.e., multireference computations with intersystem crossing rates having their origin in relativistic quantum mechanics). Thus, parts of the potential energy surfaces (PES) must be described very accurately, which consumes a lot of effort and, consequently, time. Very recently, the rapid formation of HOM from α-pinene, the most studied HOM-forming system, was uncovered by a more detailed look on the same ozonolysis-derived intermediates that had been already considered several times.^65,69,75^ Specifically it was the improved description of the excess energy partitioned into the postozonolysis vinoxy radical intermediate that was required for uncovering this important oxidation pathway.^65^

As the experimental speciation and quantification of individual HOM compounds will remain extremely challenging, the role of computations will likely only increase in the future. Especially of importance for the applied CIMS methodologies are the studies that associate reagent ion and target binding energies with instrument sensitivity^63,64^ and the use of these with controlled fragmentation to obtain experimental constraints.^76^ This also amply highlights the general need for joint theoretical-experimental approaches, as parts of the problem are easier to address computationally, whereas other areas are easier to resolve in laboratory settings. The synergistic application of both is likely to be the most fruitful one, in which theory is used to guide the experimental design, and experimental results are used to constrain the further theoretical argumentation. For the time being, computations will likely remain as the most reliable window we have on the structures of these highly functionalized gas-phase aerosol precursors.

## Challenges in Pathways

**Figure 4:**
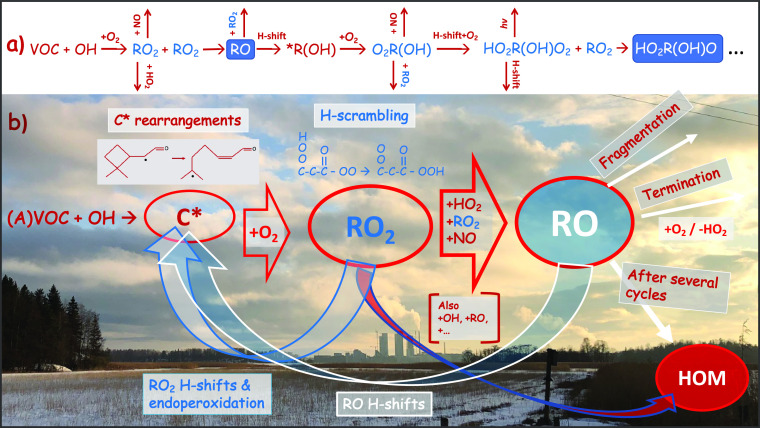
A sketch of the complex reaction pathways initiated by a single OH reaction. (a) A schematic description of the first reaction steps that propagate the oxidation chain, together with (b) a sketch of the interconnections of the reaction network. After the oxidation initiation cross reactions of the intermediate RO_*x*_ radicals (=RO and RO_2_) and reactions with NO_*x*_, (=NO and NO_2_) and HO_*x*_ (=OH and HO_2_) all complicate the ultimate outcome. The detailed branching in the pathways is dependent on the exact molecular structures of the reacting partners and on the prevailing experimental conditions. Peroxy radicals were marked in blue, alkoxy radicals are in white with a blue background, and carbon-centered radicals have an asterisk.

The biggest obstacle in describing HOM formation in any VOC system lies in the rapidly growing amount of potential branching pathways. These happen both in the (pseudo-) unimolecular oxidation propagation and in the cross combination of practically all the radical intermediates involved (see the sketch of the complexity in Figure 4). The term autoxidation stands for “autocatalytic oxidation” and is inherently a self-catalyzed phenomena.^11,77^ The gained oxidized functionalities help the subsequent hydrogen abstraction reactions (i.e., H shifts) and thus enable a propagation of the oxidation chain. With each added oxygen, the several sites of potential H abstractions become more equal, increase the possibility for isomeric reaction channels, and directly relate to the mutual experimental-theoretical challenge of determining the HOM-forming pathways and subsequent product structures. The fast radical rearrangements that do not transform the product chemical composition but only tweak the spatial structure occur very rapidly, and they are not easily tracked by the available research methodologies.

Tightly linked to the combinatorial problem has been the lack of a genuine chemical imagination. Before the pioneering theoretical work by Vereecken et al.^78^ invoking the possibility of polyoxygenates in a rapid atmospheric oxidation, and the separate field experiments and subsequent lab works by Ehn et al.^13,14^ that describe HOM for the first time as a common constituent of the ambient gas phase, practically no one thought such polyperoxide, gas-phase compounds could exist. Yet, it had been noted several times that organic compounds likely have an important role in the atmospheric particulate matter formation and growth of ambient aerosols. This was already clear by considering the mass balance of atmospheric molecules and, later, from measurements by aerosol mass spectrometers.^79^

More details on HOM compositions and a solid hypothesis for the chemical nature and formation mechanism of these compositions was soon reported by Ehn and co-workers.^10^ Subsequent studies confirmed these hypotheses and refined the formation pathways through RO_2_ autoxidation.^11,12^ The mechanism was guided by low-temperature autoignition research^80^ and a recent work by Crounse et al.^77^ However, the important difference between the autoignition and Crounse works was that the oxidation was shown to propagate into far higher oxidized intermediates and products and did not terminate the oxidation by the common **H**-C(-OOH) abstraction and subsequent ketohydroperoxide formation. Importantly, in autoignition the loosening of the C–H bonds and subsequent H-abstraction happens due to heat, whereas in an atmospheric autoxidation the loosening is brought about by nearby functional groups. Very recently Wang et al. showed how these effects can be additive and, thus, how the heat can be used to initiate autoxidation in naked alkanes too, providing a tangible segue between these two phenomenological VOC oxidation regimes.^33^ While the previous low-temperature combustion research could not observe the HOM formation due to pertaining instrumental limitations, it is likely that considerable insight could still be gained from a revisit to the extensive ignition delay and cool flame literature, potentially providing important experimental and theoretical constraints.^80^

**Figure 5:**
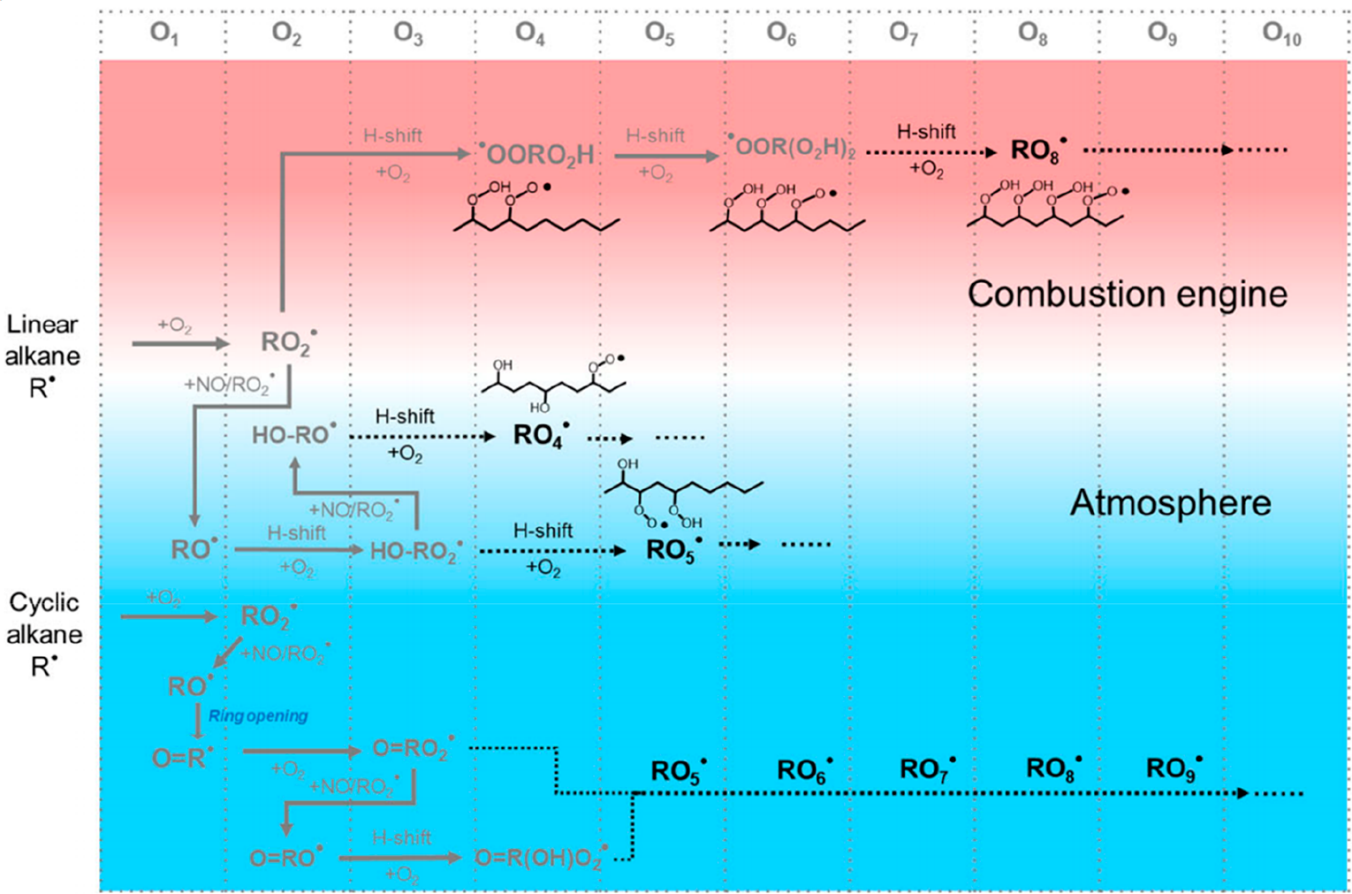
Proposed general HOM-forming pathways in alkane autoxidation. Note the special role of RO radicals in propagating the oxidation sequence. Reprinted with permission from ref (33). Copyright 2021 Springer Nature.

Thus, whether unimolecular or bimolecular, branching constitutes the major challenge in describing autoxidation of (A)VOCs. Its involvement is clear already from the first principles in considering the radical intermediates propagating the sequence, but unfortunately it is a known complexity we have not afforded to dwell into yet. The sophisticated theoretical computations have simply been too resource-demanding, and the mass spectrometric detection methodologies have not been able to follow distinct reaction pathways. Thus instead the HOM research has focused on finding a single, theoretically sound oxidation pathway able to explain a certain prominent HOM formation. Nevertheless, it has been clear from the beginning that other pathways must contribute and lead into isomeric HOM products. Recently, Noziere and Vereecken^36^ reported how even the naked alkanes have the potential for H-shift reactions, but they are too slow to provide a significant competition for other RO_2_ loss processes. Thus, bimolecular RO_2_ reactions yielding RO (e.g., with NO,^81^ RO_2_, or even HO_2_,^82^ even multiple consecutive^33^) seem pivotal for the initiation of alkane autoxidation and formation of HOM (Figure 5).

The conversions between RO_2_ and RO radicals are at the heart of AVOC autoxidation (Figure 5). When RO_2_-mediated isomerizations (i.e., H shifts and endoperoxidation) and carbon-centered radical (C*) rearrangements are inaccessible, alkoxy radicals are needed to propagate the oxidation sequence. RO undergo much more rapid H-shift reactions than RO_2_, and their more energetic nature makes for more varied branching pathways.^83^ Alkoxy radicals are also prone to decompose, and their special role in the breaking of carbon rings that hinder HOM formation has been noted previously.^39,75^ Especially in the naked, straight-chain, unbranched alkanes the role of RO is heightened, as the parent compounds do not have feasible RO_2_ hydrogen-shift reactions available.^33,36^ Alkoxy radicals are even indirectly related to the molecular mechanism of peroxide “dimer” formation by RO_2_ + RO_2_ reactions, in which two ROs will stay entangled before the O_2_ breaks apart and the peroxide −C-O-O-C– bridge is formed.^72^ Very recently alkoxy radicals were also shown to undergo bimolecular reactions even with another (stabile) radical species such as RO_2_,^84^ which highlights the poor understanding we have of their importance for the atmospheric particulate matter formation and on atmospheric chemistry in general.

The HOM and linked SOA research are rapidly approaching a status where lumped rate coefficients and simplified mechanistic rules do not suffice to explain the phenomena at the required detail. Very recently McFiggans et al.^85^ compared this to the point in which ozone-pollution research was some decades ago, that is, the details do matter. Yet this common practice of lumping in atmospheric and combustion chemistry modeling is likely still useful for several analysis, but for ambient pollutant formation and removal, especially relating to particulate matter formation, a number of molecules and oxidation processes must be considered individually. With relatively complicated atmospheric molecules such as mono- and sesquiterpenes this constitutes a considerable task in considering the wide variety of possible reaction pathways. Fortunately, the number of species likely involved in the formation of most of the atmospheric SOA is far more limited than the whole VOC pool, and a thorough knowledge of some tens of atmospheric systems is probably enough to adequately estimate the aerosol-forming potential of most gas-phase environments. Likewise, the pool of small atmospheric radicals that react with these aerosol prestages is dominated by a handful of species, decreasing the amount of interactions that must be explicitly accounted for. Yet, even this reduced number of chemical systems results in a large number of reactions and even larger number of products to consider, and thus sophisticated machine-learning methodologies or other similar approaches can hopefully soon provide order to this apparent chaos.

## Additional Unknowns—The Darker Side

Several mechanistic aspects can be envisioned to influence the likelihood of AVOC HOM formation, yet their relevance remains unclear and prevents a determination of their ultimate importance. Among the most significant uncertainties are (i) competition for oxidation initiation and propagation, (ii) role of hydrogen scrambling, (iii) role of catalytic and photolytic reaction steps, (iv) role of differing RO_2_ reactivity, and (v) role of apparent minor channels. This listing is not meant to be exhaustive and surely excludes several other burning issues that remain to be resolved.

The competition between oxidation pathways begins from the initiation of the oxidation chain starting from the primary radical production rate, whether it is by photolysis or by reactions with oxidants (e.g., competition for OH). The OH abstraction rate depends on the chemical neighborhood, and practically every C–H abstraction in a molecule will occur at a different rate, with only certain abstractions leading to subsequent HOM formation. The following steps are likewise prone for a similar competition, and even with the same primary radical production rate the following R + O_2_ propagation rates can vary significantly. However, under atmospheric conditions nearly all O_2_ reactions are fast due to oxygen being in a very large excess, which is commonly exploited in theoretical computations by omitting their calculation. Nevertheless, even the O_2_ addition rates can vary by several orders of magnitude (e.g., CH_3_CHNH_2_ + O_2_ at 5.5 × 10^–11^ cm^3^ s^–1^, while C_3_H_5_ + O_2_ at 1.6 × 10^–13^ cm^3^ s^–1^ under practically identical low-pressure reaction conditions^86,87^), and in certain cases this additional complexity could be important to account for. This competition for the oxidation propagation continues throughout the whole HOM formation sequence and is of special importance for each radical intermediate. The competition between different RO_2_ H-shift sites is likely the most determining factor in HOM formation and currently very poorly constrained, as only a few RO_2_ H-shift rates have been inspected experimentally (see, e.g., refs (19) and (43)).

The H-scrambling reactions in the oxidized radicals (e.g., H-shift between hydroperoxide and peroxy radical functional groups^88,89^) can severely complicate the assessment of correct pathways. It leaves no marks to the transformed product chemical composition and is generally a very fast reaction step. Previously it was noted to yield peroxy acids at the expense of hydroperoxides, yet in the cyclohexene ozonolysis system an NO_2_ addition to the reaction mixture apparently leads to the formation of highly oxygenated acylperoxynitrates.^81^ Mechanistically the H-scrambling reaction is very intriguing, as it can in a sense reverse the course of the reaction and return the radical site to its previous location.

The role of oxidation propagation, or even initiation, by catalytic pathways is currently poorly understood. Recently Monge-Palacios et al.^90^ showed how formic acid can catalyze the conversion of stabilized Criegee intermediates to vinylhydroperoxides (VHP), enabling a reinitiation of the oxidation sequence. Similarly the role of photolysis in inducing HOM formation has not been assessed. The photolysis of several oxidation products,^91^ even RO_2_ radicals,^92^ could provide an alternative route to the HOM formation. The aromatics and their derivatives provide an exceptionally good example, as the absorption spectra of the first- and second-generation oxidation products shift to progressively longer wavelengths and higher absorption cross sections, facilitating their photolysis under a solar illumination.^91^ However, the concentration of every new product generation is likely to be lower than that of the parent, and thus the influence is likely to stay minor. Nonetheless, this could prove to be a significant source, for example, during early mornings when solar irradiance quickly increases and the accumulated processed material from the previous night photolyzes.^93^

In any real-life VOC oxidation system the cross reactions of different RO_2_ leading to oxidation propagation and termination will have a special importance, with the rates of common RO_2_ + R′O_2_ reactions spanning many orders of magnitude.^94^ Especially for the AVOC HOM formation the RO involvement seems critical (Figures 4 and 5). Even HO_2_ reactions, which are generally waived by the implication that they only lead to ROOH, can contribute to the RO budget, and in the need for a truly realistic HOM formation description, they must be taken into account.^82^ Critical are also the branches to minor channels (e.g., minor H abstraction pathway to produce a resonance-stabilized radical in competition with a ring closure by endoperoxidation) and will remain difficult to account for. These are problematic, especially in cases where the rest of the potential energy surface of the minor channel would be far more attractive for an HOM formation but is ruled out during an early inspection of the mechanism due to computational limitations, as generally to find even one pathway with rigorous treatments is a laborious task.

The overarching realization is that HOM with a usual 0.1–10% yield generally fit into uncertainties of the previous studies. Thus, finding out the small differences in the pathways that enable HOM formation will remain very challenging but is likely to be the key in finding the HOM from the representative AVOCs. Furthermore, without significant leaps in detection and computational methodologies, it will remain very difficult to ascertain the importance of these less-clear oxidation sequences. Nevertheless, with the recent surge on the computational method development that exploits machine- and deep-learning strategies enabled by ever-increasing computing power, the needed theoretical advancements do not seem that science fiction anymore. Nonetheless, the experimental techniques have more real-life physical limitations to overcome and, thus, are likely to lag behind. Time will show.

## Outlook

While the decade of work on HOM formation by atmospheric autoxidation has increased our understanding of complex organic oxidation in the gas phase, several dark spots still exist and prevent us from describing the formation of HOM from the diversity of AVOC. The lessons learned about the likelihood of H-shift reactions are directly transferrable between BVOC and AVOC, yet far more experimental-theoretical studies are required to solidly anchor the underlying chemistry. As a topical example, also the autoxidation of the first- and second-generation reaction products must be studied (i.e., classical oxidation potentially leading to autoxidation), not the least by remembering that the H-shift reactions are faster in the oxidized intermediates than in the naked parent hydrocarbons. Currently this has been only briefly inspected by Wang et al.^33^ and Garmash et al.^39^

The rapid reaction sequence propagation by alkoxy radicals and carbon-centered radical (C*) rearrangements are likely of high importance for an accurate description of the reaction mechanisms but lack methodology for their experimental inspection. The commonly applied low-pressure and low-O_2_ environments in VOC oxidation research have a high potential to skew the results by limiting the collision frequency and, in considering the real atmosphere, collisions with the correct coreactants. The theoretical RO structure activity relationships (SAR) by Vereecken et al.^83,95^ are a good starting point in estimating the relevance of alkoxy radicals, whereas apparently nothing similar exists for the C*. While SAR are a common and practical tool in atmospheric chemistry research, such simplifications are also a big part of the reason why it took such a long time to find the pathways to HOM in the first place. In considering ambient HOM formation, we are still far away from any useful SAR development, and thus system-specific treatments are a must. Ultimately, this research will evolve enough to allow for an SAR development, but when, and how, is still too early to say.
